# APP Function and Lipids: A Bidirectional Link

**DOI:** 10.3389/fnmol.2017.00063

**Published:** 2017-03-10

**Authors:** Marcus O. W. Grimm, Janine Mett, Heike S. Grimm, Tobias Hartmann

**Affiliations:** ^1^Experimental Neurology, Saarland UniversityHomburg/Saar, Germany; ^2^Neurodegeneration and Neurobiology, Saarland UniversityHomburg/Saar, Germany; ^3^Deutsches Institut für DemenzPrävention (DIDP), Saarland UniversityHomburg/Saar, Germany

**Keywords:** lipids, APP processing, AICD, Abeta cholesterol, sphingolipids, PUFA, sulfatides, gangliosides

## Abstract

Extracellular neuritic plaques, composed of aggregated amyloid-β (Aβ) peptides, are one of the major histopathological hallmarks of Alzheimer’s disease (AD), a progressive, irreversible neurodegenerative disorder and the most common cause of dementia in the elderly. One of the most prominent risk factor for sporadic AD, carrying one or two aberrant copies of the apolipoprotein E (ApoE) ε4 alleles, closely links AD to lipids. Further, several lipid classes and fatty acids have been reported to be changed in the brain of AD-affected individuals. Interestingly, the observed lipid changes in the brain seem not only to be a consequence of the disease but also modulate Aβ generation. In line with these observations, protective lipids being able to decrease Aβ generation and also potential negative lipids in respect to AD were identified. Mechanistically, Aβ peptides are generated by sequential proteolytic processing of the amyloid precursor protein (APP) by β- and γ-secretase. The α-secretase appears to compete with β-secretase for the initial cleavage of APP, preventing Aβ production. All APP-cleaving secretases as well as APP are transmembrane proteins, further illustrating the impact of lipids on Aβ generation. Beside the pathological impact of Aβ, accumulating evidence suggests that Aβ and the APP intracellular domain (AICD) play an important role in regulating lipid homeostasis, either by direct effects or by affecting gene expression or protein stability of enzymes involved in the *de novo* synthesis of different lipid classes. This review summarizes the current literature addressing the complex bidirectional link between lipids and AD and APP processing including lipid alterations found in AD *post mortem* brains, lipids that alter APP processing and the physiological functions of Aβ and AICD in the regulation of several lipid metabolism pathways.

## Alzheimer’S Disease

Worldwide currently there are more than 46 million people suffering from dementia and the number of affected individuals is estimated to double every 20 years. Alzheimer’s disease (AD) is a devastating neurodegenerative disorder, which is the most common cause of dementia in the elderly population. Clinically AD is characterized by a progressive loss of cognitive brain functions leading to memory dysfunction, impaired judgment, disorientation and finally to a total loss of memory and personality (Plassman et al., 2007; World Alzheimer Report, 2015). AD-patients typically die in average within 3–10 years after diagnosis due to secondary disorders (Zanetti et al., 2009). The clinical symptoms of AD might be caused by an extensive loss of synapses and neurons leading to a strong hippocampal and cortical atrophy (Scheff and Price, 1993; Gómez-Isla et al., 1996; Mouton et al., 1998; Dickerson et al., 2001). The characteristic neuropathological hallmarks of the disease are intracellular neurofibrillary tangles (NFTs) and extracellular localized amyloid plaques. While the NFTs are composed of the microtubuli-associated protein tau in a hyperphosphorylated state (Grundke-Iqbal et al., 1986a,b), the amyloid plaques are mainly built up of amyloid-β (Aβ) peptides. Aβ-peptides are hydrophobic, 38–43 amino acid long products generated by the sequential proteolytic processing of the amyloid precursor protein (APP; Glenner and Wong, 1984; Masters et al., 1985; Kang et al., 1987). The significant cerebral accumulation of Aβ, starting several years prior to the first symptoms, is respected to trigger the disease process (Glenner and Wong, 1984; Glenner, 1989; Hardy and Higgins, 1992; Hardy and Selkoe, 2002). Especially the accumulation of Aβ42 (indicating 42 amino acids), which is the major Aβ species found in neuritic plaques, is considered to initiate AD progression (Iwatsubo et al., 1994; Tamaoka et al., 1995). Due to the additional hydrophobic amino acids isoleucine and alanine Aβ42 has a higher tendency to aggregate compared to the more prevalent Aβ40 (indicating 40 amino acids; Jarrett et al., 1993). Increasing evidence suggests small oligomers of Aβ to represent the most toxic form of the peptide (Lambert et al., 1998; Lesné et al., 2006; Shankar et al., 2008). Several mechanisms are discussed to contribute to Aβ neurotoxicity, among them the induction of inflammatory processes, a disruption of calcium homeostasis and membrane integrity, cholinergic and mitochondrial dysfunction and increased oxidative stress (Grimm and Hartmann, 2012).

There are two forms of AD, the more common sporadic AD with a disease onset after the age of 65 (late onset AD, LOAD) and the genetically based form (familial AD, FAD) with an earlier manifestation of symptoms. The two variants are basically distinguishable from each other in clinical and neuropathological terms. Less than 5% of all AD-cases belong to FAD which is caused by mutations in the genes encoding for APP and the presenilins (PS) 1 and 2, proteins involved in proteolytic APP-processing (Levy et al., 1990; Goedert et al., 1994; Levy-Lahad et al., 1995; Sherrington et al., 1995; Tanzi, 2012). Besides aging, hypercholesterolemia, hypertension, atherosclerosis, homocysteinemia, diabetes mellitus and obesity are discussed as non-genetic risk factors for LOAD (Barnes and Yaffe, 2011; Polidori et al., 2012). The ε4 allele of the apolipoprotein E (ApoE) has been identified as the most important genetic risk factor for the sporadic form of the disease (Corder et al., 1993; Strittmatter et al., 1993).

**Figure 1:**
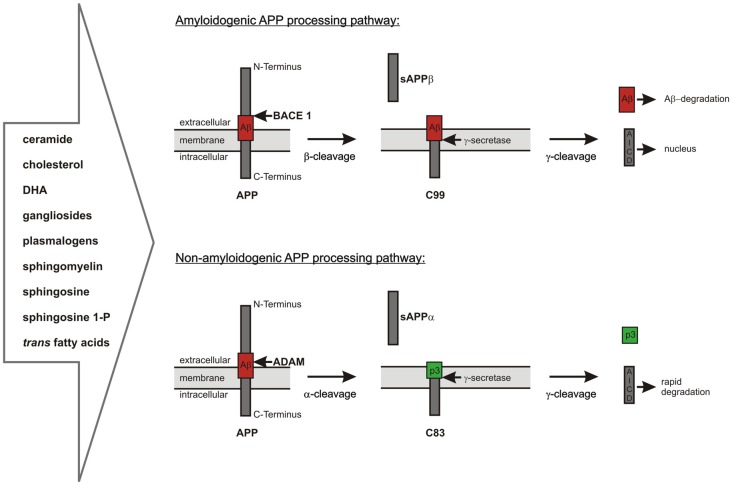
**Overview of the two alternative amyloid precursor protein (APP) processing pathways, which are highly influenced by lipid homeostasis.** Amyloidogenic pathway: APP is first cleaved by the β-secretase β-site APP cleaving enzyme 1 (BACE1) resulting in the release of sAPPβ and the generation of C99, which is further processed by the γ-secretase complex to amyloid-β (Aβ)-peptides and the APP intracellular domain (AICD). The neurotoxic Aβ-peptides can be cleared by different mechanisms including enzymatic degradation. The intracellular AICD-domain is known to translocate into the nucleus and to regulate the transcription of several target genes. Non-amyloidogenic pathway: APP is cleaved by the α-secretases belonging to the ADAM protein family within the Aβ-domain. This results in the release of sAPPα into the extracellular space and the formation of C83. C83 is further processed by the γ-secretase complex resulting in the release of the non-toxic peptide p3 into the extracellular space and of AICD into the cytosol. In contrast to the AICD generated by amyloidogenic APP processing the AICD derived from α-/γ-secretase-dependent APP processing is rapidly degraded in the cytosol and transcriptionally inactive.

As already mentioned, Aβ is generated by proteolytic processing of the precursor protein APP. APP is a ubiquitously expressed type I-transmembrane protein cycling between the plasma membrane and acidic intracellular compartments (Haass et al., 1992; Koo and Squazzo, 1994; Thinakaran and Koo, 2008). It consists of a large ectodomain, a single transmembrane domain and a short intracellular part. APP belongs to an evolutionary conserved protein family including the APP-like proteins 1 and 2 (APLP1, APLP2) in mammals. APP can be sequentially cleaved via two different pathways (Haass et al., 1992; Thinakaran and Koo, 2008; De Strooper, 2010; Figure 1). In the predominant non-amyloidogenic processing pathway the generation of Aβ is precluded. It is initiated by the α-secretase dependent cleavage of APP within the Aβ-domain shedding off the soluble ectodomain sAPPα and generating the membrane-anchored C-terminal fragment (CTF) C83 (indicating 83 amino acids). Members of the ADAM (a disintegrin and metalloprotease) protein family have been identified as catalytically active α-secretases with ADAM10 representing the physiologically relevant, constitutive α-secretase in neurons (Lammich et al., 1999; Kuhn et al., 2010). In contrast, the aspartyl protease β-site APP cleaving enzyme 1 (BACE1) initiates the amyloidogenic APP-processing pathway generating the membrane-spanning CTF C99 (indicating 99 amino acids) and releasing sAPPβ into the extracellular space (Vassar et al., 1999). The two alternative pathways differ in their subcellular localization: due to the acidic pH-optimum of BACE1 the amyloidogenic APP-processing is localized in acidic intracellular compartments, while non-amyloidogenic APP-processing mainly takes place at the cell surface (Parvathy et al., 1999; Grbovic et al., 2003; Carey et al., 2005). In both pathways the CTFs are subsequently processed by the γ-secretase complex, which consists of the proteins PS1 or PS2 as the catalytic core, Aph1 (anterior pharynx defective 1) a or b, PEN2 (presenilin enhancer 2) and nicastrin (Baulac et al., 2003; Edbauer et al., 2003; Kimberly et al., 2003). The γ-secretase possesses the unusual property to cleave its substrates within their transmembrane domains after shedding off the ectodomain, a process called regulated intramembrane proteolysis (RIP; Brown et al., 2000; Lichtenthaler et al., 2011). This catalytic activity leads to the generation of the non-toxic peptide p3 out of C83 and of Aβ out of C99 combined with the release of APP intracellular domain (AICD) into the cytosol in both processing pathways (Passer et al., 2000; Kakuda et al., 2006; Grimm and Hartmann, 2012). Due to multiple γ-secretase cleavage sites within the transmembrane domain of APP, the generated Aβ- and AICD-peptides can vary in length (Funamoto et al., 2004; Qi-Takahara et al., 2005; Kakuda et al., 2006). AICD is reported to translocate to the nucleus and to regulate the transcription of target genes, among them the genes encoding for APP, BACE1, the Aβ-degrading protease neprilysin (NEP) as well as several enzymes involved in lipid metabolism (Cao and Südhof, 2001; von Rotz et al., 2004; Grimm et al., 2011b,d, 2012c, 2013, 2015a).

## Link Between Lipids and AD

A link between AD pathology and lipids was already observed more than a century ago by Alois Alzheimer, who described a higher occurrence of “adipose inclusions” or “lipoid granules” in *post mortem* AD-brain tissue as a third pathological hallmark of the disease (Foley, 2010). In the meantime the content of several lipid classes and fatty acids has been found to be altered in the brain of AD-patients. A physiological function of Aβ and AICD in the regulation of several lipid metabolism pathways has been reported, possibly explaining the altered cerebral content of some lipid species in AD-affected brain tissue. Inversely, APP-processing is strongly influenced by the surrounding lipid environment indicating a bidirectional link between APP-proteolysis and lipid metabolism (Grimm et al., 2012b; Mett et al., 2014).

The link between lipid homeostasis and AD-pathology is strengthened by the identification of the ApoEε4-allele as the most important genetic risk factor for LOAD. ApoE is a lipoprotein involved in the transport of cholesterol and other lipids in the central nervous system (CNS). In humans there are three different ApoEε alleles encoding for the isoforms ApoEε2, ApoEε3 and ApoEε4 (Weisgraber et al., 1981; Mahley et al., 1996; Holtzman et al., 2012). The ApoEε4-allele is associated with an increased AD-risk, earlier disease onset and enhanced cerebral plaque load (Corder et al., 1993; Kuusisto et al., 1994; Breitner et al., 1999; Tiraboschi et al., 2004). In contrast, ApoEε2-carriers have a reduced risk of developing AD (Corder et al., 1994). These associations might be explained by an isoform-dependent binding of ApoEε (ε2 > ε3 > ε4) to Aβ-peptides influencing the clearance and aggregation of the peptide (Ma et al., 1994; Deane et al., 2008; Castellano et al., 2011; Holtzman et al., 2012).

A strong impact of the surrounding lipid bilayer on APP-processing is given by the fact that APP as well as all secretases are transmembrane proteins and that γ-secretase dependent APP cleavage even takes place in the hydrophobic membrane environment. For example, the exact position of γ-secretase cleavage and hence the length of the generated Aβ-peptides depends on membrane thickness (Grziwa et al., 2003; Winkler et al., 2012). In addition, the membrane fluidity influences APP-processing. Increased membrane fluidity seems to stimulate the non-amyloidogenic APP-processing by reducing APP internalization (Kojro et al., 2001). In this context it is important to note that APP-processing is also influenced by the subcompartmentalization of the membrane. Lipid raft microdomains are compact, dynamic assemblies of membrane proteins enriched in cholesterol, gangliosides and other sphingolipids. They are detergent-resistent and strongly differ in their lipid composition from the surrounding non-raft domains. Implications of lipid rafts in the intracellular protein trafficking, protein-lipid and protein-protein interactions as well as transmembrane signaling have been reported (Brown and Rose, 1992; Lingwood and Simons, 2010). The generation of Aβ has been shown to mainly take place in lipid rafts due to the co-localization of APP with BACE1 and the γ-secretase complex within these membrane microdomains (Lee et al., 1998; Riddell et al., 2001; Ehehalt et al., 2003; Vetrivel et al., 2004). In contrast, the non-amyloidogenic APP-proteolysis seems to occur predominantly in non-raft regions (Ehehalt et al., 2003; Harris et al., 2009). All these details indicate that a modulation of the membrane lipid composition might provide the opportunity of influencing Aβ-generation.

In the following sections of this article, the impact of several lipids and fatty acids on Aβ-associated AD-pathology is reviewed as well as the regulation of the corresponding metabolic pathways by APP-processing.

## The Impact of Cholesterol on AD

The brain is the most cholesterol-rich organ in the body (23 mg/g), it contains 23% of the total body sterol while only accounting for 2.1% of the total body weight (Dietschy and Turley, 2004). Within brain tissue cholesterol is mainly present in myelin sheaths and in the membranes of glial cells and neurons in its unesterified form. Due to the limited transport of cholesterol across the blood-brain barrier the cerebral cholesterol level is mainly dependent on *de novo* synthesis by oligodendrocytes, astrocytes and to a lesser extent by neurons. The conversion of 3-hydroxy-3-methylglutaryl-CoA to mevalonate catalyzed by the hydroxymethylglutaryl-CoA reductase (HMGCR), which is inhibited by statins, is the rate-controlling step in cholesterol biosynthesis (Martins et al., 2009; Di Paolo and Kim, 2011). The first evidence for a link between AD-pathogenesis and cholesterol metabolism was provided in 1994 by the observation that dietary cholesterol increases Aβ-production in rabbits (Sparks et al., 1994). Today there are many lines of evidence arguing for a connection between the pathology of AD and cholesterol homeostasis which are summarized below.

In several epidemiological studies elevated serum/plasma cholesterol contents have been identified as a risk factor for developing AD. Especially high serum cholesterol level in midlife are associated with a higher AD-risk (Pappolla et al., 2003; Solomon et al., 2009; Matsuzaki et al., 2011; Meng et al., 2014). Additionally, enhanced level of low-density lipoprotein (LDL) cholesterol and reduced level of high-density lipoprotein (HDL) cholesterol in serum correlate with the cerebral amyloid deposition in living human beings (Reed et al., 2014). In line, in human *post mortem* AD-brains cholesterol was found to be elevated and highly enriched in amyloid plaques (Cutler et al., 2004; Xiong et al., 2008; Panchal et al., 2010).

Most cell culture studies revealed that increasing cellular cholesterol level lead to an enhanced Aβ production whereas a depletion or reduction of cholesterol by e.g., cyclodextrin or statins shows the opposite effect (Simons et al., 1998; Fassbender et al., 2001; Maulik et al., 2013). The Aβ increasing property of cholesterol is based on a direct activation of β- and γ-secretase proteolytic activity (Kalvodova et al., 2005; Grimm et al., 2008; Osenkowski et al., 2008). Cholesterol is enriched in lipid raft membrane microdomains, in which amyloidogenic APP-processing mainly takes place. Thus modulating cellular cholesterol content inevitably affects membrane structure, membrane fluidity as well as cellular processes associated with lipid raft microdomains. Cholesterol depletion leads to the disruption of lipid rafts and therefore to a reduced association of APP, BACE1 and the components of the γ-secretase complex to lipid raft membrane microdomains, resulting in decreased amyloidogenic APP processing. Vice versa, an increase of cellular cholesterol leads to a higher lipid raft content of the membranes and hence to Aβ-overproduction (Simons et al., 1998; Hao et al., 2001; Hicks et al., 2012). High membrane cholesterol levels additionally promote APP endocytosis leading to enhanced Aβ-production in acidic intracellular compartments (Cossec et al., 2010). Conversely, APP is primarily localized at the cell surface in cholesterol-depleted cells leading to increased α-secretase-dependent non-amyloidogenic APP processing (Kojro et al., 2001). Beside the cholesterol-mediated effects on APP-proteolytic processing, cholesterol has been shown to promote Aβ-aggregation and -toxicity (Schneider et al., 2006; Ferrera et al., 2008; Abramov et al., 2011).

A strong correlation between hypercholesterolemia and enhanced Aβ level has also been observed in several animal models (Sparks et al., 1994; Refolo et al., 2000; Maulik et al., 2013). Inversely, a reduction of accumulated Aβ-peptides along with improved behavioral memory was achieved in animal models after administration of cholesterol-lowering drugs including statins (Fassbender et al., 2001; Refolo et al., 2001; Kurata et al., 2012). It should be noted, that there are also a few studies in which statins had no or oppositional effects on the cerebral Aβ-content *in vivo* (Park et al., 2003; Cibickova et al., 2009).

The impact of statins on AD has also been analyzed in observational studies and randomized controlled trials leading to inhomogeneous results. Statin intake is associated with a reduced incidence of AD or dementia in general in most, but not all of these studies (Wolozin et al., 2000, 2007; Rea et al., 2005; Arvanitakis et al., 2008; Haag et al., 2009). Especially the reduction of serum cholesterol level by the intake of statins in midlife might have a preventive effect towards the development of AD (Kivipelto et al., 2002; Pappolla et al., 2003; Shinohara et al., 2014). In strong contrast, most clinical trials failed to observe any benefit of statins in individuals already suffering from AD (Feldman et al., 2010; Sano et al., 2011; McGuinness et al., 2014), indicating cholesterol-lowering drugs to have rather a protective than a therapeutic potential in respect to AD.

Beside the described influence of cholesterol on APP-proteolysis, there is also an impact of APP-processing on cholesterol homeostasis. APP/APLP2- and PS1/PS2-deficient fibroblasts have a significantly increased cellular cholesterol content, which can be reversed by the supplementation of Aβ40-peptides. In line with this, enhanced cerebral cholesterol concentrations were found in APP- and PS-deficient mice (Grimm et al., 2005; Umeda et al., 2010). Analysis of the underlying mechanisms revealed that Aβ40 reduces cholesterol *de novo* synthesis by inhibiting HMGCR activity (Grimm et al., 2005).

### Summary

The existence of a regulatory feedback cycle, in which Aβ-production is stimulated by cholesterol while cholesterol *de novo* synthesis is inhibited by high cellular Aβ40-concentrations is indicated.

### Future Directions

The heterogeneous results of studies analyzing the impact of statins on the incidence of AD denote the existence of responders and non-responders. For the future it will be important to find biomarkes to identify patients that might profit from statins.

## The Impact of Docosahexaenoic Acid (DHA) on AD

Docosahexaenoic acid (DHA, 22:6) is a polyunsaturated fatty acid (PUFA) naturally occurring in high amounts in marine food, especially in fish oil (Mann et al., 2010). It accounts for 30%–40% of all esterified fatty acids in neuronal plasma membrane phospholipids and for 8% of the brain dry weight, thus belonging together with α-linolenic acid (ALA, 18:3) and eicosapentaenoic acid (EPA, 20:5) to the most important ω3-fatty acids in the CNS (Lauritzen et al., 2001; Muskiet et al., 2006). As endogenous DHA-biosynthesis is highly limited in humans, the main part of this fatty acid is provided by dietary intake (Pawlosky et al., 2001). DHA is efficiently transported across the blood brain barrier (Ouellet et al., 2009; Nguyen et al., 2014) and rapidly incorporates into phospholipids of cellular membranes leading to increased membrane fluidity (Horrocks and Farooqui, 2004; Yang et al., 2011).

The DHA content is reported to be reduced in the serum/plasma of AD-patients as well as in certain regions of *post mortem* AD-brains (Söderberg et al., 1991; Conquer et al., 2000; Tully et al., 2003). Because of its six double-bonds DHA is very susceptible to lipid-peroxidation resulting in oxidative stress known to be involved in AD pathogenesis (Smith et al., 1994; Yatin et al., 1998; Fam et al., 2002; Cai et al., 2011). Indeed, the levels of PUFA oxidation products are elevated in AD-affected brains, indicating the reduced DHA content in these tissues to be caused by increased oxidative damage (Sayre et al., 1997; Markesbery and Lovell, 1998; Montine and Morrow, 2005; Grimm et al., 2016a).

Several epidemiological trials found the dietary intake of DHA or higher DHA serum/plasma levels to be associated with a reduced risk of developing AD indicating a potential of DHA in AD-prevention (Kalmijn et al., 1997; Barberger-Gateau et al., 2002; Morris et al., 2003b). However, other studies failed to find an association between PUFAs and AD-risk (Engelhart et al., 2002; Kröger et al., 2009; Jicha and Markesbery, 2010; Mett et al., 2014).

We and others analyzed the impact of DHA on APP-processing revealing the fatty acid to reduce Aβ-levels via pleiotropic mechanisms. DHA reduces β- and γ-secretase activity and stimulates α-secretase-dependent APP-cleavage. In addition to direct effects, the activities of γ- and β-secretase are reduced by DHA due to a PS1-displacement out of lipid rafts and a reduced BACE1 internalization. The stimulated α-secretase activity in presence of DHA is based on the enhanced gene expression and protein stability of ADAM17. Altogether these effects lead to a shift from amyloidogenic to non-amyloidogenic APP-processing and thus to reduced total Aβ-level. DHA additionally has cholesterol-lowering effects further inhibiting Aβ-production. It reduces cholesterol *de novo* synthesis via inhibition of HMGCR and disturbs lipid raft integrity by shifting cholesterol out of these membrane microdomains (Hashimoto et al., 2005a; Stillwell et al., 2005; Grimm et al., 2011c). Beside the described effects on APP-processing an impact of DHA on Aβ-degradation and -aggregation has also been reported. We recently observed a highly enhanced insulin-degrading enzyme (IDE)-dependent Aβ-degradation in neuroblastoma cells after the supplementation of DHA- and EPA-containing phosphatidylcholine (PC; Grimm et al., 2016b). Others reported an increased microglial phagocytosis of Aβ as well as a reduction of Aβ-fibrillation and Aβ-induced toxicity in the presence of DHA (Hossain et al., 2009; Hjorth et al., 2013).

A protective effect of dietary DHA with regard to cerebral Aβ-level and amyloid plaque load could be further confirmed *in vivo* in several animal models (Lim et al., 2005; Green et al., 2007; Perez et al., 2010). In line with this, higher cognitive performances were observed in AD-animal models after DHA supplementation (Hashimoto et al., 2002, 2005b; Calon et al., 2004). However, others failed to find any beneficial effect of DHA in AD transgenic mice (Arendash et al., 2007).

A possible therapeutic use of DHA regarding AD has been investigated in several clinical trials showing inconsistent results. Some studies revealed a beneficial effect of daily DHA treatment in patients with very mild cognitive dysfunctions (Freund-Levi et al., 2006; Kotani et al., 2006; Chiu et al., 2008). Others did not observe any influence of DHA on AD-biomarkers and cognitive decline in AD patients (Freund-Levi et al., 2009; Quinn et al., 2010). It should be mentioned, that oxidized DHA species and the lipid-peroxidation products of PUFAs are able to increase amyloidogenic APP-processing and hence Aβ-generation. In a recent study we demonstrated, that only 1% oxidized DHA reverts the positives effects of DHA on Aβ-production indicating that PUFAs have to be prevented from oxidation in nutritional approaches (Grimm et al., 2016a). In such approaches DHA often is combined with E-vitamins due to their high antioxidative properties acting as scavengers of radicals and peroxides (Kamal-Eldin and Appelqvist, 1996). However, we demonstrated in two recent studies that several tocopherol and tocotrienol species have beside their protective antioxidative properties the undesirable effect of increasing amyloidogenic APP processing and reducing the enzymatic degradation of Aβ-peptides (Grimm et al., 2015b, 2016c).

### Summary

Despite the inhomogeneous results of clinical studies there are several epidemiological and molecular indications for a beneficial effect of DHA in preventing AD and possibly halting its progression, at least at very early disease stages. The fact that its oxidation products are able to reverse the beneficial effects of DHA might partially explain the divergent outcomes of clinical DHA studies and underlines the need to prevent DHA from oxidation in such trials.

### Future Directions

Because of the controversial effects of several E-vitamins regarding the molecular mechanisms of AD, the identification of further molecules for the prevention of DHA from oxidative damage in nutritional approaches without side effects on APP processing might be valuable. Additionally, the combination of DHA with precursors/cofactors for membrane synthesis and synaptogenesis as for example uridine-monophosphate, choline and phospholipids might further strengthen its beneficial effects on cognition as demonstrated in a transgenic mouse model of AD (Koivisto et al., 2014).

## The Impact of *Trans* Fatty Acids on AD

*Trans* fatty acids (TFAs) are unsaturated fatty acids, which are characterized by having at least one double-bond in *trans*-configuration. This means that the two hydrogen atoms are, in contrast to *cis-*configuration, localized on opposite sides of the double-bond. Because of their straighter shape compared to the *cis*-counterparts, TFAs have higher melting points and lead to a decreased fluidity of biological membranes (Roach et al., 2004; Ibrahim et al., 2005). TFAs in our diet arise from industrial procedures and to a lesser extent from biological processes in the digestive tract of ruminant animals. The key source of TFAs is commercially prepared food due to hydrogenation or thermal treatment of oils (Bhardwaj et al., 2011). Accumulation of these fatty acids in the body as well as incorporation in brain tissue has been reported indicating an impact of TFAs on cerebral biochemistry (Laryea et al., 1990; Teixeira et al., 2012).

Studies analyzing the relationship between TFAs and AD-risk or the progression of cognitive decline came to inconsistent results. A positive correlation between dietary TFA intake and AD-risk was found in one study while others reported the AD-risk not to be influenced by TFAs (Engelhart et al., 2002; Morris et al., 2003a). Similarly, some authors observed the TFA intake to result in a higher rate of cognitive decline in women with type 2 diabetes, in persons with high copper consumption and in older persons in general while others failed to find a relationship between TFA intake and cognitive decline in women (Morris et al., 2004, 2006; Devore et al., 2009; Naqvi et al., 2011; Okereke et al., 2012).

We investigated the effects of TFAs on APP-processing and Aβ-generation in neuroblastoma cells compared to their *cis*-counterparts. In presence of TFAs, we found a shift from non-amyloidogenic to amyloidogenic APP-processing accompanied by a significant increase in Aβ-production. TFA supplementation increases the activity of β- and γ-secretase due to direct effects and an enhanced gene expression of BACE1 and the γ-secretase complex components (Grimm et al., 2012a). The direct effect on γ-secretase activity was confirmed by others demonstrating the activity of purified γ-secretase to be stimulated by an increased *trans/cis*-ratio of supplemented fatty acids (Holmes et al., 2012). In contrast, non-amyloidogenic APP-processing is reduced in TFA-treated cells because of enhanced APP-internalization and a reduction in ADAM10 gene expression. Additionally, we found TFAs to stimulate Aβ-aggregation *in vitro* (Grimm et al., 2012a).

The impact of TFAs on cerebral Aβ-levels and cognition has also been investigated *in vivo* with less clear results. In a study by Phivilay et al. (2009), Aβ- and tau-pathology was unaltered in the brain tissue of an AD-mouse model after dietary supplementation of TFAs. Another study reported a declined spatial learning performance of mice fed with a TFA- and monosodium glutamate-rich diet (Collison et al., 2010).

As TFAs are reported to be linked to cholesterol and DHA homeostasis they might also affect APP-processing and Aβ-generation via indirect mechanisms. The dietary intake of TFA leads to an inauspicious enhanced ratio of LDL/HDL plasma cholesterol (Mensink and Katan, 1990; Judd et al., 1994), which might be associated with a higher AD-risk as described above. Furthermore, high TFA consumption was shown to modify the fatty acid profile of murine brain tissue with a reduction in DHA content. Nevertheless, in this study the cerebral Aβ-levels were unaltered as already mentioned (Phivilay et al., 2009).

### Summary

Due to the dissimilar results of studies analyzing the impact of TFAs on AD-risk and Aβ-associated pathology *in vivo*, further trials are necessary to clarify the role of these fatty acids in AD-pathogenesis.

### Future Directions

If the negative effects of TFA on AD-risk can be confirmed *in vivo*, a stronger reduction of TFA intake should be recommended, particularly because of the accumulation of these fatty acids in the human body over time and their incorporation into brain tissue (Laryea et al., 1990; Teixeira et al., 2012).

## The Impact of Plasmalogens on AD

Plasmalogens (PL) are commonly occurring phospholipids accounting for 22% of the total phospholipid mass in human brain tissue. They are characterized by an enol ether double-bond at the sn1-position, which links an alkenyl chain to the glycerol backbone. At the sn2-position they are enriched in PUFAs including DHA and arachidonic acid (AA, 20:4). Phosphatidylethanolamine (PE) and PC are the most common polar head groups of PLs, which have a high susceptibility to oxidative stress due to their enol ether double-bond (Broniec et al., 2011; Braverman and Moser, 2012). PL-biosynthesis takes place in peroxisomes and the endoplasmic reticulum. The initial committed step reaction of PL *de novo* synthesis is catalyzed by the peroxisomal enzyme alkyl-dihydroxyacetonephosphate-synthase (AGPS; De Vet et al., 1999). PL level in the human body are mainly modulated by PL metabolism, but to a lesser extent also by the dietary consumption of PL-rich meat and fish (Blank et al., 1992).

While one study by Pettegrew et al. (2001) did not detect an AD-dependent alteration in the cerebral PL-content, we and others found a reduction of PE-PLs and PC-PLs in human *post mortem* AD-brains (Ginsberg et al., 1995; Han et al., 2001; Grimm et al., 2011a; Igarashi et al., 2011; Rothhaar et al., 2012). In line with this, a reduced PE-PL content was also observed in the serum and in erythrocyte membranes of AD-patients (Goodenowe et al., 2007; Oma et al., 2012).

The reduction of PL content in AD-affected brain tissue might be explained by enhanced PL degradation due to increased oxidative stress and a stimulated activity of phospholipases in presence of Aβ-peptides (Sanchez-Mejia et al., 2008). Additionally, we demonstrated PL biosynthesis to be regulated by APP-processing. Under physiological conditions AGPS gene expression and hence PL biosynthesis is upregulated by AICD. In contrast, under pathological conditions the Aβ-induced reactive oxidative species impair AGPS protein stability leading to a decreased PL *de novo* synthesis (Grimm et al., 2011d).

Because of the altered PL content in AD-brain tissue, we analyzed the impact of PLs on APP-processing. Our results demonstrate that PLs reduce γ-secretase activity in living cells as well as in purified membranes derived from neuroblastoma cells and murine brain tissue. Compared to the corresponding phospholipids lacking the enol ether, all tested PC-PL- and PE-PL-species independent of the bound fatty acid directly inhibited γ-secretase activity. In contrast, the activities of α- and β-secretase remained unchanged after PL-supplementation (Rothhaar et al., 2012). The direct inhibitory effect of PE-PLs on the γ-secretase complex has been recently confirmed by others (Onodera et al., 2015). Interestingly, in our study the addition of PLs to cellular membranes derived from human AD-brains also resulted in a decreased γ-secretase activity. This indicates the rebuilding of a normal PL level to have a positive impact in the pathologic situation of AD (Rothhaar et al., 2012). However, such *ex vivo* experiments have their clear limitations and further studies are necessary to analyze the *in vivo* relevance of PLs on APP-processing. In addition to Aβ-production, there is also an impact of PLs on the aggregation of Aβ-peptides. PE-PL has been reported to eliminate the neurotoxicity-associated Aβ-oligomerization phase while allowing fibril formation (Lee et al., 2011).

### Summary

In the pathologic situation of AD a vicious cycle between PLs and Aβ-generation can be postulated: the accumulation of Aβ results in a reduced cerebral PL content stimulating γ-secretase activity and hence leading to a further increased Aβ-production.

### Future Directions

In the future the *in vivo*-relevance of the effects of PLs on the generation of Aβ-peptides should be analyzed. An interesting human model for such trials could be cells derived from patients affected by Zellweger syndrome, which show deficient PL-levels due to a defective peroxisome assembly (Styger et al., 2002; Saitoh et al., 2009).

## The Impact of Sphingolipids on AD

Sphingolipids are an inhomogeneous group of lipids characterized by a backbone consisting of the amino alcohol sphingosine. Sphingolipid biosynthesis is initiated by the serine palmitoyl-CoA transferase (SPT) catalyzing the condensation of palmitoyl-CoA and L-serine to 3-ketosphinganin, which is further metabolized to ceramide. Ceramide is the most important branching point within the sphingolipid metabolism pathways serving as precursor for the generation of sphingosine, sphingomyelin (SM) and more complex glycosphingolipids.

All sphingolipids are anchored in the membrane bilayer via their ceramide moiety, besides cholesterol they represent major components of lipid raft membrane microdomains (Posse de Chaves and Sipione, 2010). The first evidence for a role of sphingolipids in neurodegeneration came from the observation of lysosomal storage diseases, inherited disorders characterized by the lysosomal accumulation of different sphingolipids. These diseases are associated with early dementia and the development of AD-related Aβ- and tau-pathology (Tarasiuk et al., 2012). The link between sphingolipid metabolism and AD-pathogenesis is further strengthened by alterations of several sphingolipids in *post mortem* AD-brain tissue and their potential to modulate APP-processing and Aβ-aggregation summarized below. Additionally, SPT gene expression and hence total sphingolipid biosynthesis is downregulated by the APP-processing product AICD (Grimm et al., 2011b).

### Ceramide

As already mentioned, ceramide is generated by *de novo* synthesis and by hydrolysis of various more complex sphingolipids. Ceramides are pro-apoptotic and neurotoxic signaling molecules, additionally participating in the regulation of cellular proliferation and differentiation (Dawson et al., 1998; Toman et al., 2002).

The ceramide level has been reported to be increased in different brain regions and in the cerebrospinal fluid (CSF) of AD-patients. As the increase in ceramide content is already present at the earliest clinical stages of AD, it might be speculated that it is involved in disease development (Han et al., 2002; Satoi et al., 2005; Katsel et al., 2007; He et al., 2010; Filippov et al., 2012). Such a relationship is supported by a 9-year-follow-up study reporting an association between elevated baseline serum ceramide levels and an enhanced risk for developing AD (Mielke et al., 2012).

As reported by Katsel et al. (2007) the accumulation of ceramide in AD-affected individuals might be explained by multiple gene expression abnormalities. The authors found an increased cerebral expression of genes involved in ceramide *de novo* synthesis along with a reduced expression of genes required for glycosphingolipid formation out of ceramide. Another explanation for the increased ceramide content in AD-brain tissue is the Aβ-mediated activation of sphingomyelinases (SMases) catalyzing the brake down of SM to ceramide. We and others found Aβ-peptides to directly stimulate neutral SMase (nSMase)-activity (Jana and Pahan, 2004; Lee et al., 2004; Grimm et al., 2005), a stimulation of acidic SMase (aSMase) by Aβ has also been observed (Malaplate-Armand et al., 2006). The resulting enhanced ceramide level is reported to be a mediator of Aβ-induced apoptosis. Besides a probable involvement in Aβ-induced cell death, ceramide also affects APP-cleavage. Accumulation of endogenous ceramide levels in cultured cells by the use of cell-permeable C6-ceramide or by nSMase treatment promotes amyloidogenic APP-processing. The resulting ceramide-induced enhanced Aβ biogenesis is caused by a post-translational stabilization of the β-secretase BACE1 due to elevated acetylation of the protein (Puglielli et al., 2003; Ko and Puglielli, 2009).

In their entirety these facts indicate the existence of a feed-forward cycle between ceramide and Aβ under the pathological conditions in AD-brain tissue: enhanced ceramide level lead to an increased Aβ-production resulting in the activation of SMases and hence in a further elevation of ceramide content, which stimulates Aβ-production and might be involved in the induction of apoptotic cell death.

### Sphingomyelin

SM accounts for approximately 10% of mammalian cellular lipids and is highly enriched in myelin sheets. It is produced out of ceramide by the activity of SM-synthases, SMases catalyze the catabolic break down of SM back to ceramide.

The already mentioned increased ceramide content and the upregulation of SMases in *post mortem* AD brains (Katsel et al., 2007; He et al., 2010) suggests that SM concentrations might be reduced in these tissues. However, the results of studies analyzing the SM content in AD-affected brains are inhomogenous (Pettegrew et al., 2001; Cutler et al., 2004; Bandaru et al., 2009; He et al., 2010). In addition, SM level were found to be significantly increased in the CSF of individuals with prodromal AD while there was a slight, but not significant reduction of SM in the CSF of patients with mild and moderate AD (Kosicek et al., 2012). In an epidemiological study by Mielke et al. (2011) higher SM concentrations and an enhanced SM/ceramide-ratio in plasma was found to correlate with a decelerated disease progression among AD-patients.

In strong contrast to ceramide, SM was demonstrated to inhibit Aβ-production. Increasing SM content of cultured cells either by direct exposure or nSMase inhibition leads to a significant decrease of Aβ-peptides caused by an inhibition of γ-secretase dependent APP-processing. In this study we additionally identified the already mentioned direct stimulation of nSMase by Aβ42 (Grimm et al., 2005).

Accordingly, the Aβ-induced elevation of SMase-activity in AD-brain tissue results in an enhanced ceramide/SM-ratio. The increase in γ-secretase activity due to lowered SM-level in combination with the ceramide-dependent activation of β-secretase further promotes Aβ-production might result in a futile cycle.

### Sphingosine and Sphingosine 1-Phosphate

Ceramidases catalyze the conversion of ceramide to sphingosine, which is phosphorylated by sphingosine kinase (SK) generating the anti-apoptotic and neuroprotective molecule sphingosine 1-phosphate (S1P). S1P has been demonstrated to induce cell survival and proliferation and to antagonize Aβ- and ceramide-induced cell death (Cuvillier et al., 1996; Gomez-Brouchet et al., 2007; Czubowicz and Strosznajder, 2014). In contrast, sphingosine seems to have a role in apoptosis, cooperatively or independently from ceramide signaling (Sweeney et al., 1998; Lepine et al., 2004).

In line with an increased acid ceramidase expression and activity, the sphingosine content has been found to be elevated in *post mortem* AD-brains (Huang et al., 2004; He et al., 2010). It should be mentioned, that there is also another study reporting a decreased acid ceramidase gene expression in AD-brain tissue (Katsel et al., 2007). In contrast, the cerebral S1P-content seems to be declined in AD-affected individuals and to negatively correlate with the level of Aβ and phosphorylated tau protein (He et al., 2010). In line with these observations, γ-secretase activity is reduced in cells devoid of S1P-lyase degrading intracellular S1P (Karaca et al., 2014). Contrariwise, S1P has been shown to increase the production of Aβ-peptides by directly stimulating β-secretase activity in another study (Takasugi et al., 2011). Therefore, further studies are necessary to clarify the role of sphingosine and S1P in APP-processing and AD-pathogenesis.

### Sulfatides

Sulfatides are complex glycosphingolipids generated from ceramide by the addition of a galactose moiety and a sulfate group catalyzed by ceramide galactosyltransferase (CGT) and cerebrosidesulfotransferase (CST), respectively. They are highly enriched in myelin sheaths and mainly synthesized by oligodendrocytes.

Several studies reported the cerebral sulfatide content to be dramatically decreased in AD-patients compared to cognitive normal controls. These alterations were already observed in the earliest recognizable states of the disease (Han et al., 2002; Bandaru et al., 2009; Cheng et al., 2013). However, there are two other studies which failed to find a significant alteration in sulfatide content in AD-brain tissue (Cutler et al., 2004; Chan et al., 2012). CSF sulfatide level are also strongly reduced in AD-patients as reported by Han et al. (2003b) who suggested the sulfatide/phosphatidylinositol ratio in the CSF to be a potential AD-biomarker.

Interestingly, there seems to be a link between sulfatide homeostasis and ApoE: sulfatides are associated with ApoE-containing particles in the CSF and ApoE is involved in the modulation of cellular sulfatide content in an isoform-dependent manner. This possibly provides an explanation for the genetic association between ApoE and AD (Han, 2010). A role of ApoE in the regulation of cerebral sulfatide level has been demonstrated by Cheng et al. (2010). In this study the age-dependent decline in cortical sulfatide concentrations of APP transgenic mice was found to be totally abolished in ApoE-knockout animals. The sulfatide content in murine brain tissue was further demonstrated to be dependent on ApoE-genotype. In comparison to human ApoEε3 and wildtype ApoEε, the human ApoEε4-isoform is associated with a strong sulfatide depletion in the brain of transgenic mice (Han et al., 2003a). Additionally, sulfatides seem to be involved in ApoE-dependent Aβ-clearance. Treatment of cultured cells with sulfatides results in a strong reduction of Aβ-peptides in the culture media. The underlying mechanism was identified as a facilitated ApoE-mediated Aβ-clearance through an endocytotic pathway in response to elevated sulfatide levels (Zeng and Han, 2008).

Their robust depletion in *post mortem* AD-brain tissue and their potential to strongly reduce Aβ-levels *in vitro* indicate that sulfatides might be an attractive target in AD research. Further studies are necessary to investigate the role of this lipid class in the molecular mechanisms of the disease.

### Gangliosides

Gangliosides, sialic acid containing glycosphingolipids, represent 6% of the total lipid content in brain. They are abundant in the luminal leaflet of cellular organelles and the outer leaflet of the plasma membrane, where they are localized in lipid raft microdomains. Important functions of gangliosides in the development, proliferation and differentiation of neuronal cells have been reported. The glycosylceramide synthase (GCS) catalyzes the first step of ganglioside biosynthesis by glycosylating ceramide. Dependent of the number of sialic acid residues gangliosides are classified into four catagories, the *o*-, *a*-, *b*- and *c*-series. In brain tissue the most common gangliosides are GM1, GD1a, GD1b and GT1b belonging to the *a*- and *b*-series. GM3 is the precursor of all *a*- and *b*-series gangliosides, which are segregated by the GD3-synthase (GD3S)-catalyzed addition of sialic acid to GM3 (Busam and Decker, 1986; Lahiri and Futerman, 2007; Yu et al., 2011).

In AD-brain tissue there is a reduction of total ganglioside content along with significant regional differences in the distribution of specific ganglioside species. In brains affected by FAD and LOAD the total ganglioside level is decreased in several brain regions (Kalanj et al., 1991; Svennerholm and Gottfries, 1994; Gottfries et al., 1996). Kracun et al. (1991) reported a reduction of all major brain gangliosides combined with an increase in the more simple GM2 and GM3 in the cortex of AD-patients. In line with this, the GM1 and GM2 level were found to be elevated in the lipid raft fraction derived from cortical regions of AD brains (Molander-Melin et al., 2005). In summary, in AD-affected brains complex gangliosides tend to decrease while there is an elevation of simple ganglioside species.

Interestingly, in *post mortem* AD-brains GM1 and GD1a have been found to be associated with Aβ-plaques forming GAβ-complexes exhibiting early pathological changes of AD. This indicates a role of these ganglioside species in Aβ-aggregation (Nishinaka et al., 1993; Yanagisawa et al., 1995). Indeed, GM1 induces a conformational transition of Aβ from random coil to β-sheet structure and triggers the formation of toxic Aβ-fibrils (Choo-Smith et al., 1997; Hayashi et al., 2004; Okada et al., 2007). Further studies demonstrated an accumulation and aggregation of Aβ in GM1-enriched lipid rafts leading to an increased cytotoxicity (Wakabayashi et al., 2005).

Besides Aβ-aggregation, APP-processing and hence Aβ-generation is also influenced by GM1 and other gangliosides. Direct administration of total ganglioside extract to purified γ-secretase leads to an enhanced enzyme activity and increases the ratio of generated Aβ42 to Aβ40 peptides (Holmes et al., 2012). In line, the inhibition of GCS and hence total ganglioside biosynthesis results in a significant reduction of Aβ-production in various cell lines. The addition of exogenous brain gangliosides reverses these effects indicating the reduction of total ganglioside biosynthesis to be beneficial in AD. In this study, the authors found glycosphingolipids to affect APP-processing via regulating the subcellular APP-transport in the secretory pathway (Tamboli et al., 2005). In our own study we demonstrated GCS gene expression to be regulated by PS and APP. Deficiency in these proteins or the inhibition of γ-secretase activity results in an increased GCS gene expression and hence in increased glycosylceramide and total ganglioside level *in vitro* and *in vivo*. We showed that GCS is upregulated in the brain tissue of an AD-mouse model and of patients suffering from LOAD. Accordingly, total ganglioside *de novo synthesis* is modulated by APP-processing and deregulated in the pathological situation of AD (Grimm et al., 2014).

The treatment of neuroblastoma cells with GM1 has been shown to stimulate Aβ-generation and to reduce the sAPPα level without affecting sAPPβ (Zha et al., 2004). In strong contrast to this, peripheral injections of GM1 reduce the cerebral Aβ-burden in an AD-mouse model, possibly due to the promotion of Aβ-degradation in the periphery (Matsuoka et al., 2003). In another study the impact of GD3S deficiency, which results in a loss of *b*-series gangliosides and an accumulation of GM3, GM1 and GD1a, on the cerebral Aβ-levels in an AD-mouse model has been analyzed. Compared to the control animals, the GD3S-depleted mice showed an almost completely eliminated Aβ-associated neuropathology and no cognitive decline (Bernardo et al., 2009). In line with this, we found the generation of Aβ in cultured cells to be reduced after GM3 supplementation while the addition of the GD3S-product GD3 stimulated Aβ-release. In this context it is important to mention that we also found a regulation of GD3S by APP-processing. The activity of GD3S is inhibited by a direct interaction of Aβ with GM3 leading to a reduced substrate availability and hence to an impaired conversion of GM3 to GD3. Additionally, the gene expression of GD3S is downregulated by AICD. These results indicate the existence of a regulatory feedback cycle, in which Aβ and AICD increase the GM3/GD3-ratio leading to a reduction of amyloidogenic APP-processing (Grimm et al., 2012c).

All these data indicate a strong link between ganglioside homeostasis and AD. As the single ganglioside species differ in their amyloidogenic potential, further studies are necessary to identify the most promising molecular target in ganglioside metabolism for developing therapeutic approaches regarding AD.

### Summary

In *post mortem* AD-brain tissue there are alterations in the content of several sphingolipid species, which can be partially explained by an impact of Aβ and AICD on enzymes involved in sphingolipid homeostasis. Several sphingolipid classes have been shown to affect the proteolytic processing of APP and Aβ-clearance: ceramides, total gangliosides, GM1 and GD3 are associated with an increased Aβ-level while SM, sulfatides and GM3 have the opposite effect.

### Future Directions

The fact that ceramide is associated with an increased amyloidogenic APP processing while an increase in SM-levels results in a decreased Aβ-generation indicates SMases to be interesting pharmacological targets regarding AD. Hence, the impact of SMase-inhibitors as for example fluoxetine, maprotiline or desipramine (Kölzer et al., 2004; Kornhuber et al., 2008) on the proteolytic processing of APP and on cognitive functions should be analyzed in suitable models. Another molecular target might be the GD3S, whose inhibition results in an enhanced GM3/GD3-ratio leading to a reduction in amyloidogenic APP proteolysis. In this context it should be mentioned, that mice lacking the GD3 synthase gene show abnormalities in the sciatic nerve and in peripheral nerve regeneration along with impaired neurogenesis and behavioral deficits (Ribeiro-Resende et al., 2014; Wang et al., 2014). This phenotype indicates that pharmacological interventions in ganglioside homeostasis might be associated with severe side effects.

## Lipids as Potential Biomarkers for AD

Regarding therapeutic interventions for AD an early diagnosis of the disease and hence the identification of biomarkers, which can be used for the *in vivo* diagnosis prior to the first symptoms, is important. So, the identification of early AD-biomarkers with a high specificity and reliability is a central topic in AD research (Fiandaca et al., 2014). The lipid alterations connected to AD, which are partially detectable at the very early disease stages as described above, might have the potential to be used as biomarkers for early AD diagnosis by lipidomic approaches. For example, Mapstone et al. (2014) discovered a set of eight PC species and two acylcarnitines in the peripheral blood that predicts the development of mild cognitive impairment or AD within 2–3 years with an accuracy of more than 90%. However, further studies are needed to identify combinations of lipidomics-based biomarkers which can be used for the detection of preclinical AD with the required sensitivity and specificity.

## Conclusion

**Figure 2:**
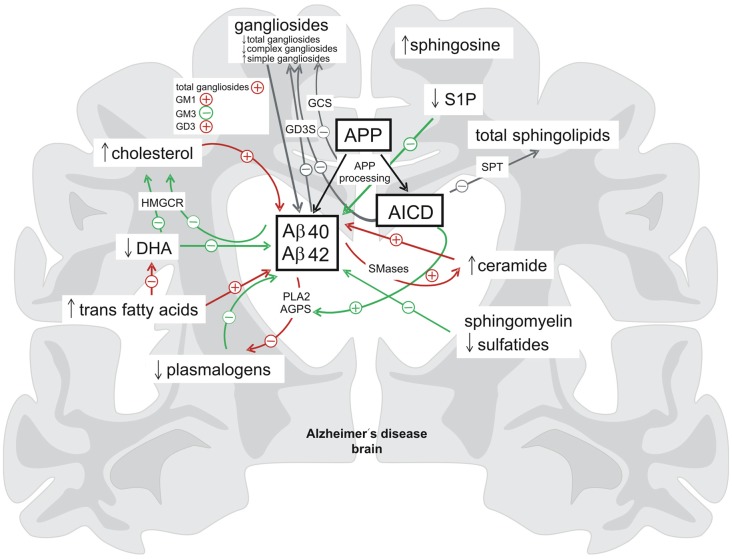
**Summary of the bidirectional link between proteolytic processing of the APP and lipid homeostasis.** In brain tissue affected by Alzheimer’s disease (AD) the levels of several lipid classes and fatty acids are altered (indicated by ↓ = decreased, ↑ = increased). Lipids and fatty acids have a strong impact on the cerebral Aβ-levels and there is also a regulation of lipid homeostasis by the APP-processing products Aβ and AICD (delineated by + = increasing effect, − = decreasing effect) indicating the existence of complex regulatory cycles between lipid homeostasis and proteolytic APP processing (green arrows = beneficial effects, red arrows = negative effects, gray arrows = neutral/unknown effects). AGPS, alkyl-dihydroxyacetonephosphate-synthase; DHA, docosahexaenoic acid; GCS, glycosylceramide synthase; GD3S, GD3-synthase; HMGCR, hydroxymethylglutaryl-CoA reductase; PLA2, phospholipase A2; S1P, sphingosine 1-phosphate; SMases, sphingomyelinases; SPT, the serine palmitoyl-CoA transferase.

In conclusion all these findings demonstrate a close link of APP, APP processing and AD to lipid homeostasis. It could be demonstrated that APP processing and especially AICD has a physiological function in in the regulation of several lipid metabolic pathways. Inversely, APP-processing is strongly dependent on the lipid microenvironment indicating a bidirectional link between APP-proteolysis and lipid metabolism. This results in tightly connected complex regulatory cycles (Figure 2). Under pathological situations such as AD, this balanced regulation might be disrupted leading to pathological alterations in lipid homeostasis and Aβ peptide overproduction, resulting in increased neurodegeneration.

## Author Contributions

MOWG, JM, HSG and TH wrote the manuscript.

## Funding

According to the author guidelines, funding for the research leading to these results were received from: the EU FP7 project LipiDiDiet, Grant Agreement No. 211696. Moreover funding for MOWG and TH was provided by Fundació la Maratò de TV3 and by JPND MindAD 1ED1508.

## Conflict of Interest Statement

The authors declare that the research was conducted in the absence of any commercial or financial relationships that could be construed as a potential conflict of interest.
